# Development of TaqMan^® ^MGB fluorescent real-time PCR assay for the detection of anatid herpesvirus 1

**DOI:** 10.1186/1743-422X-6-71

**Published:** 2009-06-04

**Authors:** Yufei Guo, Anchun Cheng, Mingshu Wang, Chanjuan Shen, Renyong Jia, Shun Chen, Na Zhang

**Affiliations:** 1 Avian Disease Research Center, College of Veterinary Medicine, Sichuan Agricultural University, Yaan 625014, PR China; 2 Key Laboratory of Animal Diseases and Human Health of Sichuan Province, Sichuan Agricultural University, Yaan 625014, PR China

## Abstract

**Background:**

Anatid herpesvirus 1 (AHV-1) is an alphaherpesvirus associated with latent infection and mortality in ducks and geese and is currently affecting the world-wide waterfowl production severely. Here we describe a fluorescent quantitative real-time PCR (FQ-PCR) method developed for fast measurement of AHV-1 DNA based on TaqMan MGB technology.

**Results:**

The detection limit of the assay was 1 × 10^1 ^standard DNA copies, with a sensitivity of 2 logs higher than that of the conventional gel-based PCR assay targeting the same gene. The real-time PCR was reproducible, as shown by satisfactory low intra-assay and inter-assay coefficients of variation.

**Conclusion:**

The high sensitivity, specificity, simplicity and reproducibility of the AHV-1 fluorogenic PCR assay, combined with its wide dynamic range and high throughput, make this method suitable for a broad spectrum of AHV-1 etiologically related application.

## Background

China is currently holding the largest waterfowl population in the world and its waterfowl production industry has been characterized by an increasing expansion and rapid development during the past decades [1]. However, infectious diseases represent the biggest obstacle to successful development of this business. Anatid herpesvirus 1 (AHV-1) infection alternatively known as duck virus enteritis (DVE), or duck plague (DP) [2], is one of the most widespread and devastating diseases of waterfowls in the family Anatidae and has severally affected the waterfowl industry since the early 1900s because relatively high mortality could be observed and a wide host range including domestic [3] and wild ducks [4,5], geese and swans of all species as well as other birds like coots are susceptible. Furthermore, serious carcass condemnations and decreased egg production were also observed in affected waterfowls. Like other herpesvirus-induced diseases, AHV-1 infection has latent form and the virus can be persistently shed by birds that recover from the disease [6]. This complicates the control of the disease, particularly under small-holder farming conditions prevalent in China.

The causative agent of AHV-1 is grouped in the alphaherpesviridae subfamily of the herpesvirus family [7] and the viral genome is a linear, double-stranded DNA molecule approximately 180 kb in size and its structure is similar to other alphaherpesviruses [8]. The AHV-1 genomic DNA has % G + C content of 64.3, which is the highest reported for any avian herpesvirus in the alphaherpesviridae [9].

Since prevention and early detection are presently the most logical strategies for virus control, various diagnostic procedures including microscopic, immunological and molecular methods have been developed for AHV-1 detection, of which the polymerase chain reaction (PCR) is a powerful tool with exquisite sensitivity for detection of minute amounts of nucleic acids, even against a high background of unrelated nucleic acids. Fluorescent quantitative real-time PCR (FQ-PCR) technique has eliminated the need of sample post-amplification handling required by the conventional PCR assay and has paved the way towards fully automated detection systems now that they usually display very high sensitivity and broad dynamic capacity after optimization [10-12]. Since virus load and proliferation dynamics serve as indispensable indicators of virus-host interaction, antiviral evaluation, active/latent infection [13-15] and guidance for therapeutic intervention, FQ-PCR is therefore of paramount importance by its exquisite virus detection and monitoring ability [16].

The detection of AHV-1 by TaqMan real-time PCR method has only been reported by Yang [17] and with the development of technology, TaqMan Minor Groove Binding (MGB™) probes as an upgrade of the ordinary TaqMan probe has been widely used during the recent years since the following advantages: (1) The TaqMan MGB probe is characterized by the conjugation of minor groove binders which facilitates highly specific of the detection. (2) The TaqMan MGB probe contains a quencher dye that does not emit fluorescence within the detectable wavelength range and results in greater accuracy in the measurement. Therefore a TaqMan MGB-based real-time PCR method for detection and quantitation of AHV-1 is developed to serve as an alternative and improvement of the previously developed ordinary TaqMan real-time PCR method.

## Results

### Development and optimization of FQ-PCR and conventional PCR

Following the optimization of FQ-PCR, final concentrations of primers each of 0.3 μmol/L and probe of 0.1 μmol/L were selected. The MgCl_2 _concentration was balanced to 6 mM that provided optimal AHV-1 amplification. Therefore the optimized 25- μL real-time PCR reaction system for AHV-1 detection could be summarized as follows: 1 × PCR buffer, 6 mmol/L MgCl_2_, 0.2 mmol/L dNTPs, 0.3 μmol/L each primers, 0.1 μmol/L probe, 1 U Taq and 1 μL DNA template.

Following the optimization of conventional PCR, the MgCl_2 _concentration was balanced to 2.5 mM and the annealing temperature of 52°C was selected. Therefore the optimized conventional PCR reaction system could be summarized as follows: 1 × PCR buffer, 2.5 mmol/L MgCl_2_, 0.2 mmol/L dNTPs, 0.5 μmol/L each primers, 1.25 U Taq and 1 μL DNA template. The optimized annealing temperature was 52°C.

### Fluorescent quantitative real-time PCR standard curve establishment

**Figure 1:**
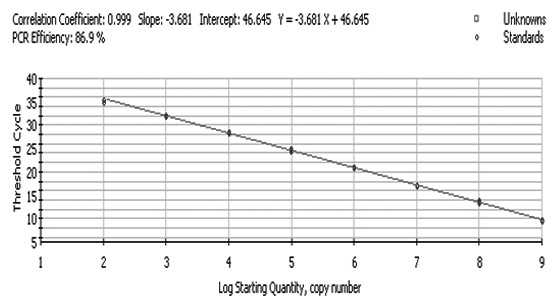
**Establishment of the fluorescent quantitative real-time PCR standard curve**. Standard curve of the AHV-1 fluorescent quantitative real-time PCR. Ten-fold dilutions of standard DNA ranging from 1.0 × 10^9 ^to 1.0 × 10^2 ^copies/μL were used, as indicated in the x-axis, whereas the corresponding cycle threshold (CT) values are presented on the y-axis. Each dot represents the result of triplicate amplification of each dilution. The correlation coefficient and the slope value of the regression curve were calculated and are indicated.

The FQ-PCR amplification curves and the corresponding fluorescent quantitative real-time PCR standard curve (Figure 1) were generated by employing the successively diluted known copy number of pAHV-1 for real-time PCR reaction under the optimized conditions. From the results of correlation coefficient (0.999) and PCR efficiency (86.9%) of the standard curve by the established FQ-PCR, it could be known that the standard curve and the established FQ-PCR are excellent at performance.

### Sensitivity, specificity, reproducibility and dynamic range of the established FQ-PCR

Different 32 AHV-1 strains kindly provided by the Avian Disease Research Center of Sichuan Agricultural University were examined with the established FQ-PCR method and these specimens all tested positive in the FQ-PCR assay, indicating that this method is sensitive and compatible with wide range of AHV-1 viruses. Ten-fold dilution series of pAHV-1 plasmid standard DNA were tested by the established real-time PCR assay to evaluate the sensitivity of the system and the detection limit was found to be 1.0 × 10^1 ^copies/reaction. Comparisons were made between conventional PCR and the established FQ-PCR using dilution series to calculate the end point sensitivity of each assay. The results indicate that the established FQ-PCR is around 100 times more sensitive than the conventional PCR method, detecting pAHV-1 down to dilutions of 1.0 × 10^1^, compared to dilutions of only 1.0 × 10^3 ^for conventional PCR.

The test using DNA from several other bacteria and viruses used as template to examine the technique's specificity showed that none of the bacteria, virus (other than AHV-1) and duck embryo fibroblast tested gave any amplification signal and the results demonstrated that the established FQ-PCR assay is of highly specific.

The intra-assay and inter-assay CV of this established FQ-PCR was in the range of 1–3% for most of the dynamic range (from 1.0 × 10^9 ^to 1.0 × 10^2 ^pAHV-1 plasmid copies/μL), but increased to more than 6% at viral DNA loads lower than 1.0 × 10^2 ^pAHV-1 plasmid copies/μL and increased to more than 4% at viral DNA loads more than 1.0 × 10^9 ^pAHV-1 plasmid copies/μL (Table 1). The results demonstrated that the established fluorescent quantitative real-time PCR method was characterized by a wide dynamic range (8 logarithmic decades) of detection from 1.0 × 10^9 ^to 1.0 × 10^2 ^pAHV-1 plasmid copies/μL with high precision. However, at lower and higher dilutions quantitation was not always reproducible compared to other properly diluted samples. Therefore the dynamic range of the method was between 1.0 × 10^9 ^and 1.0 × 10^2 ^pAHV-1 plasmid copies/μL, which is relatively broad.

**Table 1:** Intra- and inter-assay variability of the established FQ-PCR assay

Dilution of standard (copies/reaction)	Intra-assay			Inter-assay		
	Mean	SD	CV (%)	Mean	SD	CV (%)
10^10^	7.0	0.30	4.3	6.9	0.34	4.9
10^9^	9.9	0.19	1.8	10.1	0.23	2.3
10^8^	13.4	0.25	1.9	13.6	0.34	2.5
10^7^	16.9	0.29	1.7	17.0	0.36	2.1
10^6^	20.8	0.33	1.6	20.9	0.40	1.9
10^5^	24.7	0.37	1.5	24.7	0.49	2.0
10^4^	28.6	0.49	1.7	28.7	0.77	2.7
10^3^	32.3	0.52	1.6	32.2	0.90	2.8
10^2^	35.4	0.81	2.3	35.3	1.16	3.3
10^1^	37.2	2.31	6.2	37.5	2.51	6.7

### Test of established AHV-1 FQ-PCR assay using specimens for practical applications

Viral load quantification through the established AHV-1 FQ-PCR demonstrated that the AHV-1 DNA copy number of each sample could be calculated with the CT value according to the standard curve and 100% of the samples tested were quantifiable (Table 2) without the need for further sample dilution or concentration.

**Table 2:** AHV-1 viral load in different clinical samples

Sample name	DNA amounts (copies)
DEF cell culture supernatant	5.67 × 10^6^/μL
DEF cell culture	1.05 × 10^9^/μL
Allantoid fluid	2.85 × 10^6^/μL
Liver	2.73 × 10^9^/g
Brain	2.20 × 10^7^/g
Bursa of Fabricius	9.47 × 10^9^/g
Thymus	1.09 × 10^9^/g
Spleen	3.59 × 10^9^/g
Esophagus	9.56 × 10^9^/g
Duodenum	3.92 × 10^7^/g
Ileum	1.83 × 10^8^/g
Kidney	1.78 × 10^8^/g
Lung	3.15 × 10^8^/g
Peripheral blood	2.16 × 10^6^/μL
Cloacal swab	2.11 × 10^8^/swab
Oral swab	2.83 × 10^8^/swab

## Discussion

Conventional etiological, immunohistological and serological methods [18-20] were routinely used in AHV-1 identification. However, the sensitivity is usually not high enough and the methods were time-consuming since virus propagation in cell cultures is usually required and the onset of virus-induced cytopathic effect (CPE) usually requires at least 2–3 days to develop. Titration of infectious virus in cell cultures is usually achieved by the end-point dilution method in cell monolayer. Since titration of the virus load is labor-consuming and requires about 5 days for evaluation of virus-induced CPE, distinguishing between virus-induced CPE and non-specific cell alterations may be difficult, the established real-time PCR assay will be particularly suitable in these studies. In addition, an even more important factor is that the virus from tissues of infected birds is usually not readily adapted to cell culture system during the initial several rounds of propagations [21].

The PCR is a rapid, sensitive and specific nucleic acid amplification technique and many conventional qualitative PCR methods for revealing merely the presence or absence of AHV-1 pathogen have been developed and well documented [22-24]. However, the conventional PCR assays are not sufficient in a variety of clinical situations. They frequently encountered problems including the risk of cross-contamination (leading to false positives) and poor quality of extracts (leading to false negatives). Moreover, the lack of fluorogenic probes in the assay results in relative lower specificity since the amplification and detection of specific PCR products are determined solely by the amplification primers. In this paper, the development of a TaqMan MGB-based real-time PCR by using fluorogenic labels and sensitive signal detection system for detection and quantitation of AHV-1 is described. The optimized FQ-PCR detection system presented in this paper has been designed to address these issues and make it even more applicable for routine diagnostic use with several advantages over conventional PCR.

In this assay, the primers and probes have been selected on conserved DNA segments of AHV-1 genome. TaqMan Minor Groove Binding (MGB™) probes as target-specific hydrolysis oligonucleotide employed in this assay are characterized by the conjugation of minor groove binders which increases the Tm of the hybridized probe and facilitates highly specific binding to the targeted sequence [25]. Moreover this probe contains a quencher dye that does not emit fluorescence within the detectable wavelength range and results in greater accuracy in the measurement. This improvement eliminates spectral overlaps with fluorescence emitted by the reporter dye, and results in greater accuracy in the measurement of reporter-specific signals.

In view of the great sensitivity of PCR, the occurrence of false negative results is a highly underestimated problem. So an artificial construct generated by cloning of the specific target sequence into a plasmid are often used as internal controls for the amplification step. This internal positive control was incorporated into the reaction system, thus improving diagnostic conclusions, especially negative results, which is most important in the light of quarantine programs.

By carrying out direct comparisons between the established FQ-PCR method and the conventional PCR method for AHV-1 detection, the results clearly showed that overall the established FQ-PCR detection method is more sensitive and reliable when compared to conventional gel-based PCR, since it was able to detect as few as 1.0 × 10^1 ^DNA copies of template. Furthermore, this established AHV-1 FQ-PCR method shows more excellent characteristics such as dynamic range (from 1.0 × 10^9 ^to 1.0 × 10^2 ^pAHV-1 plasmid copies/μL, which is approximately 10^3 ^times broader) and sensitivity (detecting pAHV-1 plasmid down to dilutions of 1.0 × 10^1 ^copies/μL, which is about 2.3 times more sensitive) than other reported method [17].

The high quality hot start Taq DNA polymerase used in this assay could minimize unspecific amplifications and increase the PCR cycling efficiency. In addition, FQ-PCR reaction and detection is all done in a closed-tube system, the need for post-amplification manipulation is removed since the detection of the PCR products occurs online during real-time PCR amplification, hence greatly reducing the risk of cross-contamination and false positive results. The optimization of the AHV-1 FQ-PCR assay was focused on the concentration of primers and probe and Mg^2+^. When all these different practical refinements are combined, the final result is a molecular diagnostic method that is not only rapid and reliable, but one that is also easy to perform and applicable to use for testing large numbers of samples since the FQ-PCR presented the benefits of increased speed due to reduced cycle time and remove of post-amplification process, offering considerable labor savings and allowing higher throughput analysis than conventional PCR assays and thus is favorable for the transition of this method from research to routine use in laboratories. This method was preliminarily mentioned in a short report [26] but related details of primers and probe sequence, specificity test, sensitivity test, reproducibility analysis, dynamic range and internal control were unavailable. By contrast, great modification and optimization have been made in this paper to improve the quality of this study.

The AHV-1 FQ-PCR assay was highly reproducible and linear over a range of eight orders of magnitude from 10^2 ^to 10^9 ^copies, allowing a precise calculation of viral DNA load in samples containing a wide range of viral DNA amounts, eliminating the need for sample dilution and minimizing sample handling. The results for intra- and inter-assay precision indicate that both intra-assay and inter-assay CVs were satisfactorily low and the assay is reproducible, even between different batches of reagents used. Probability rather than sample quality variation is the predominant cause of variability at low copy numbers [27].

## Conclusion

In conclusion, the FQ-PCR developed in this study is highly specific and sensitive with better parameters than conventional PCR method and is a valuable method for the detection of AHV-1. The method described in this study is especially helpful for high throughput analysis such as evaluating the efficacy of antiviral drugs and experimental vaccines for AHV-1. The research group of authors is currently using this technique to study the AHV-1 distribution characteristics in vaccinated birds and in artificially infected birds. We believe that this method could expedite related AHV-1 research in the AHV-1 viral molecular biology.

## Methods

### Cell, virus and PCR template DNA preparation

Duck embryo fibroblast (DEF) monolayer was incubated at 37°C with 5% CO_2 _in tissue culture flasks with Minimal Essential Medium (MEM) that contained 10% fetal bovine serum (FBS), 100 U/mL penicillin, and 100 μg/mL streptomycin.

Anatid herpesvirus 1 (AHV-1, CHv virulent strain) was obtained from the Avian Disease Research Center of Sichuan Agricultural University (Yaan, Sichuan, China). Virus stock was added onto the surface of the cell layer which was about 90% confluency at time of infection and the maximum virus titers could usually be obtained 48 h postinfection.

DNA extraction from AHV-1 infected DEF cells and tissues of AHV-1 infected ducks were performed by using TIANamp Genomic DNA extracting kit (Tiangen Corporation, Beijing, China) according to the manufacture's instructions.

### PCR primers and probe design

The FQ-PCR assay primers and probe (named Real-F, Real-R and Real-P respectively) design was carried out using the Primer Express™ software supplied by Applied Biosystems and their sequences were listed in Table 3. The forward and reverse primers amplified a 60 bp fragment of AHV-1 DNA polymerase gene as described (GenBank Accession No. AF064639). The fluorogenic probe was labelled at 5' with FAM (6-carboxyfluorescein) dye as reporter and labelled at 3' with TAMRA (tetra-methylcarboxyrhodamine) as quencher and 3'with MGB™ (Minor Groove Binder).

**Table 3:** Oligonucleotide sequences of primers and probes used in AHV-1 FQ-PCR detection

Name	Type	Sequences (5'to 3')	Length (nt)	Position	Amplicon size (bp)
Real-F^a^	Forward	ttttcctcctcctcgctgagt	21	357–377	60
Real-P^a^	Probe	ccctgggtacaagcgc	16	383–398	
Real-R^a^	Reverse	ggccgggtttgcagaagt	18	399–416	
Con-F^b^	Forward	ggacagcgtaccacagataa	20	246–265	498
Con-R^b^	Reverse	acaaatcccaagcgtag	17	727–743	
IC-F^c^	Forward	acgagcgcaacccttga	17	1054–1070	92
IC-P^c^	Probe	cggtttgtcaccggcagtcacct	23	1103–1125	
IC-R^c^	Reverse	acgtcatccccaccttact	19	1127–1145	

^a ^Based on the nucleotide sequence AF064639.

^b ^Based on the nucleotide sequence AF064639.

^c ^Based on the nucleotide sequence AJ971894.

The conventional PCR amplification was carried out using primers designed using the Primer Premier™ software according to the sequence as described (GenBank Accession No. AF064639). The forward primer and reverse primer (named Con-F and Con-R respectively) sequences were listed in Table 3 and this primer pair yielded a 498 bp amplicon, in which the 60 bp FQ-PCR fragment was nested.

All probes and primers were synthesized by Genecore Corporation (Shanghai, China) and purified by corresponding HPLC system.

### Development and optimization of fluorescent quantitative real-time PCR and conventional PCR

The real-time PCR was carried out using the ABI AmpliTaq Gold DNA polymerase system with an icycler IQ Real-time PCR Detection System (Bio-Rad Corp., Hercules, CA) according to the manufacturer's instructions. The reaction, data acquisition and analysis were performed using iCycler IQ optical system software. The Real-time PCR was performed in an 25 μL reaction mixture containing 1 × PCR buffer, 0.2 mmol/L dNTPs, 1 U Taq and 1 μL DNA template according to the manufacture's instructions. Autoclaved nanopure water was added to bring the final volume to 25 μL. The two-step PCR cycling condition was as follows: initial denaturation and hot-start Taq DNA polymerase activation at 95°C for 10 min, 50 cycles of denaturation at 94°C for 15 s, primer annealing and extension at 60°C for 20 s with fluorescence acquisition during each annealing and extension stage. Real-time PCR reactions were optimized in triplicate based on primer, probe and MgCl_2 _concentration selection criteria, which was performed according to 4 × 4 × 4 matrix of primer concentrations (0.2, 0.3, 0.4 and 0.5 μmol/L), probe concentrations (0.05, 0.1, 0.2, and 0.3 μmol/L) and MgCl_2 _concentrations (2, 4, 6 and 8 mmol/L).

The conventional PCR was performed and optimized on a Mycycler™ thermo cycler system (Bio-Rad Corp., Hercules, CA, USA) with a 50 μL PCR reaction system containing 1 × PCR buffer, 0.2 mmol/L dNTPs mixture, 1.25 U rTaq (Takara Bio Inc., Shiga, Japan), 0.5 μmol/L each forward and reverse primers and 1 μL template DNA. All PCR experiments were carried out in 0.2 ml thin-walled tubes with the following cycle parameters: The mixture was subjected to initial denaturation at 95°C for 1 min, followed by 50 cycles of 95°C for 60 s, annealing for 60 s, extension at 72°C for 60 s, and one cycle of final extension at 72°C for 5 min. The amplified 498 bp product then underwent electrophoresis on 1.0% agarose gels. Electrophoresis was carried out at 100 V in a Mini-sub (Bio-Rad Corp., Hercules, CA, USA) gel electrophoresis unit and gels were viewed under a UV transilluminator. The conventional PCR reactions were optimized based on MgCl_2 _concentration and annealing temperature selection criteria in a similar way as that of Real-time PCR and the selection was made by the brightness of the amplified 498 bp fragments on the agarose gel under a UV transilluminator.

An internal positive control was introduced into the FQ-PCR assay to verify the absence of DNA losses during the extraction step and of PCR inhibitors in the DNA templates. The internal positive control of pGM-T recombinant vector (designed as pB16S) consisting of Bacillus 16S rRNA gene (GenBank Accession No. AJ971894) sequence amplified with primers (IC-F and IC-R) listed in Table 3 was added into the lysis buffer at the concentration of 1.0 × 10^6 ^copies/μL. Real-time PCR for IC detection was carried out in a separate run, using primers and probe (named IC-F, IC-R and IC-P respectively) listed in Table 3. The fluorogenic probe was labelled at 5' with FAM as reporter and labelled at 3' with TAMRA. The quantitative real-time PCR protocol was the same as that of AHV-1 detection. From the ratio of the calculated amount of IC to the actual amount of IC, which is shared by the specimen, the normalization could be achieved and the actual amount of AHV-1 in the specimen could be obtained. Actually this internally controlled method has been widely used in other related detection assays [28,29].

### Fluorescent quantitative real-time PCR standard curve establishment

The 498 bp conventional PCR target amplicon band on agarose gel was cut and the DNA was recovered and purified by TIANquick DNA Purification system (Tiangen Corp., Beijing, China) according to the instruction manual of the product. The product was ligated into pGM-T vector (Tiangen Corp., Beijing, China) and transformed into E.coli DH5α competent cells. Recombinant plasmid (designated as pAHV-1) was extracted using TIANprep plasmid extraction kit (Tiangen Corp., Beijing, China). Presence of the target DNA insert was confirmed by PCR amplification and sequencing.

The standard curve of the FQ-PCR was generated by successive dilutions of the known copy number of pAHV-1. Recombinant plasmid pAHV-1 concentration was determined by taking the absorbance at 260 nm using a Smartspec 3000 spectrophotometer (Bio-Rad Corp., Hercules, CA) and purity was confirmed using the 260/280 nm ratio. Through its molecular weight, pAHV-1 copy number was then calculated and the purified pAHV-1 plasmid DNA was then serially diluted 10-fold in TE buffer, pH 8.0, from 1.0 × 10^9 ^to 1.0 × 10^2 ^plasmid copies/μL. These dilutions were tested in triplicate and used as quantitation standards to construct the standard curve by plotting the plasmid copy number logarithm against the measured CT values. The Bio-Rad iCycler IQ detection software created the standard curve, calculated the correlation coefficient (R^2^) of the standard curve, standard deviations of triplicates.

### FQ-PCR sensitivity, specificity, reproducibility and dynamic range analysis

Different 32 AHV-1 strains (derived from a wide spectrum of sources, subsequently confirmed through related etiological methods, and then preserved by the Avian Disease Research Center of Sichuan Agricultural University) including virulent and avirulent strains were examined with the established FQ-PCR method to test the sensitivity and compatibility of this method. In addition, the sensitivities of the conventional PCR and FQ-PCR were each determined using triplicates of different concentrations of recombinant plasmid pAHV-1. Template DNA was prepared as follows: plasmids of pAHV-1 were diluted serially in 10-fold steps from 10^10 ^copies/μL to 10^1 ^copies/μL using sterile ultra pure water. One microliter from each dilution was used as template and subjected to the conventional PCR and FQ-PCR protocol respectively. The detection limit of the conventional PCR was determined based on the highest dilution that resulted in the presence of clear and distinct amplified fragments (498 bp) on the agarose gel. The detection limit of the FQ-PCR was determined based on the highest dilution that resulted in the presence of CT value in real-time PCR detection.

DNA from duck embryo fibroblast (DEF) and several other pathogens including duck hepatitis B virus, *Salmonella enteritidis*, duck adenovirus, goose parvovirus, Marek's disease virus, infectious laryngotracheitis virus and *Pasteurella multocida *(kindly provided by Avian Diseases Research Center of Sichuan Agricultural University) were used as templates in triplicates to confirm the technique's specificity.

Within-run and between-run reproducibilities of the FQ-PCR assay were assessed by multiple measurements of pAHV-1 samples of different concentrations. The assay was conducted by assessing the agreement between the replicates in five replicates (within-run precision) and in five separate experiments (between-run precision) of the serially diluted pAHV-1 recombinant plasmid samples through performing analysis of the mean coefficient of variation (CV) values of each AHV-1 standard dilution.

Dilutions of pAHV-1 recombinant plasmid were used to determine the dynamic ranges of the FQ-PCR assay. The lower and upper limits of quantification were defined by the pAHV-1 recombinant plasmid sample concentrations possessing reasonable precision.

### Test of established AHV-1 FQ-PCR assay using specimens for practical applications

AHV-1 infected duck embryo fibroblast culture, allantoid fluid and other specimens including liver, brain, Bursa of Fabricius, thymus, spleen, esophagus, duodenum, ileum, kidney, lung, peripheral blood each collected from AHV-1 infected ducks were employed to assess the ability of the established FQ-PCR to detect AHV-1 in a variety of usually used samples. By this assay viral load quantification was obtained.

## Competing interests

The authors declare that they have no competing interests.

## Authors' contributions

YG carried out most of the experiments and wrote the manuscript. AC and MW critically revised the manuscript and the experiment design. CS, RJ, SC and NZ helped with the experiment. All of the authors read and approved the final version of the manuscript.
